# Increasing Physical Activity and Reducing Dementia Risk in Older African American Residents of Public Housing

**DOI:** 10.1093/geroni/igaa057.1964

**Published:** 2020-12-16

**Authors:** Bernadette Fausto, Paul Duberstein, Shou-En Lu, Mark Gluck

**Affiliations:** 1 Rutgers University, Newark, New Jersey, United States; 2 Rutgers University School of Public Health, Piscataway, New Jersey, United States

## Abstract

Older African Americans—especially those with lower income and those living in urban public housing—have a greater risk of Alzheimer’s disease (AD) compared to the general population. Inadequate levels of physical activity and aerobic exercise are thought to be among the probable causes for increased AD risk. Based on our preliminary data, we hypothesize that a cluster-randomized multi-level intervention in low-income public housing, focused on heart and brain health, can produce participant-level increases in physical activity among participants enrolled in an aerobic exercise class after six months (primary outcome) that are maintained at one year, as well as housing-level changes in attitudes and beliefs about physical activity and exercise participation among housing residents, both exposed and not exposed to the participant-level intervention as well as participant-level improvements in cognition and brain health evincive of decreased risk for AD.

